# Patient-Prosthesis Mismatch in Aortic Valve Replacement With No.19 Prosthesis: Impact on Early and Late Outcomes

**DOI:** 10.7759/cureus.80121

**Published:** 2025-03-06

**Authors:** Selman Dumani, Alessia Mehmeti, Ermal Likaj, Laureta Dibra, Alfred Ibrahimi, Andi Kacani, Klodian Krakulli, Edvin Prifti, Aferdita Veseli, Stavri Llazo, Ilir Alimehmeti, Ali Refatllari, Altin Veshti

**Affiliations:** 1 Division of Cardiac Surgery, University Hospital Center "Mother Theresa", Tirana, ALB; 2 Cardiac Surgery, University Hospital Center "Mother Theresa", Tirana, ALB; 3 Anesthesiology, University Hospital Center "Mother Theresa", Tirana, ALB; 4 Cardiac Surgery, University Hospital Center "Mother Teresa", Tirana, ALB; 5 Department of Family and Occupational Health, Faculty of Medicine, University of Medicine, Tirana, ALB; 6 Health Commission, Academy of Sciences of Albania, Tirana, ALB; 7 Division of Cardiac Surgery, University Hospital Center "Mother Teresa", Tirana, ALB

**Keywords:** aortic valve, aortic valve replacement, aortic valve surgery, body surface area, indexed effective orifice area, mismatch severity, nyha class, patient-prosthesis mismatch, retrospective

## Abstract

Introduction: Patient-prosthesis mismatch (PPM) continues to be a crucial topic of discussion regarding its influence on the aortic valve surgery results. The aim of our study is to evaluate the incidence of mismatch and its influence on early and late results in aortic valve replacement using prosthesis No 19.

Methods: A cohort of 175 patients underwent aortic valve replacement with prosthesis No-19 and was followed for up to 10 years. PPM was defined according to indexed effective orifice valve area (EOAi). Early results were evaluated through hospital mortality, and the data were collected from hospital recordings. Long-term follow-up was realized by contacting the patients by phone. The endpoints were mortality and the clinical status assessed by New York Heart Association (NYHA) class change after intervention.

Results: The overall and severe mismatch incidence was 69.1% and 29.7%, respectively. Overall, hospital mortality is 3.4%. Severe mismatch (p=0.057) and continuous values of indexed effective orifice area (p = 0.079), adjusted for sex and age, do not affect early mortality. Severe mismatch and indexed effective orifice area were associated with less post-operative improvement of NYHA functional class (p<0.015) in the long term, independently from other predictors.

Conclusions: Severe mismatch and small indexed orifice areas do not affect early and late mortality, but they are related, importantly, with less symptomatic improvement in the long-term outcomes.

## Introduction

Prosthesis-patient mismatch occurs when the effective surface area of the implanted prosthesis is smaller in comparison with the normal human valve [1]. This results in the development of elevated gradients in normally functioning prostheses. It remains an actual issue during aortic valve replacement regarding the impact on early and/or late post-operative outcomes, with rates varying from 24-48% for moderate mismatch and 8-18% for severe PPM [2]. Nevertheless, the majority of research findings concur on a significant correlation: "Prosthesis-patient mismatch is linked to compromised hemodynamic performance, limited regression of left ventricular mass following prosthesis insertion, increased incidence of cardiac events, and decreased overall survival".

This phenomenon may be more common when small prostheses are implanted. The current investigation includes patients who underwent aortic valve replacement with prosthesis no. 19. The aim of this study is to identify the incidence of prosthesis-patient mismatch in this group of patients in our institution and evaluate the effects of this phenomenon on early mortality and late post-operative outcomes.

## Materials and methods

This is a retrospective study. We included 175 patients who underwent aortic valve replacement with prosthetic valve No- 19 from 1st January 2013 to 23rd December 2023. For assessing the presence of prosthesis-patient mismatch (PPM), we used this classification: Indexed effective orifice valve area (EOAi) < 0.65 cm^2^/m^2^ as severe PPM; 0.65 cm^2^/m^2^ < EOAi ≤ 0.85 cm^2^/m^2^ as moderate PMM and EOAi > 0.85 cm^2^/m^2^ as no PPM [1]. The indexed effective orifice area of the prosthetic valves was calculated using reference tables of aortic prosthetic valve production companies [3,4]. The demographic and early post-operative outcomes data were collected from patients' medical records. Early post-operative outcomes included in-hospital mortality and perioperative major complications (such as hemorrhage, respiratory failure, kidney failure, stroke etc.). Long-term post-operative results were obtained through patient surveys. The survey was conducted by phone based on a questionnaire we prepared. Patients were asked about their clinical progress. The follow-up period ranged from 1 to 9 years after intervention. We used Delta NYHA, the change in NYHA class after the intervention, as an indicator to analyze the clinical progress of the patients. Out of the initial 175 patients, only 136 of them responded to the survey to be included in the long-term follow-up results. The statistical program used was IBM SPSS Statistics for Windows, Version 21.0. The tests used to obtain the results were Chi-test, logistic regression, and linear regression. Results are expressed in odds ratio point estimate, 95% confidence interval, and p-value for the logistic regression, whereas results are expressed in beta point estimate, 95% confidence interval, and p-value for the linear regression. A p-value of <0.05 was considered statistically significant.

## Results

The study included 175 patients who underwent aortic valve replacement with a biological or mechanical prosthesis No- 19, with only 136 assessed in the long-term follow-up. The mean population's age was 63.54± 11.00, and most of them were between 61-70 years of age. The female gender occupied 72.6% of the group, and aortic valve stenosis was the predominant pathology. More than half (54.4%) of the patients were hospitalized in NYHA III clinical status. The most often simultaneous surgery was coronary artery bypass grafting.

PPM was present in 69.1% (121/175) of cases for both types of prosthesis and is more frequently related to biological prosthesis than mechanical ones: 36% vs. 33%, respectively. Meanwhile, the incidence of severe PPM was 29.7% (52/175), and the incidence of moderate PPM was 39.4% (69/175).

In the following tables, we present a detailed overview of the abovementioned results, specifically the baseline demographic and clinical data shown in Table 1 and the baseline ultrasonography data shown in Table 2.

**Table 1:** Baseline demographic and clinical data NYHA: New York Heart Association Classification

Baseline Demographic and Clinical Data	n (%)
Gender	175 (100%)
Females (%)	127 (72.6%)
Males (%)	48 (27.4%)
Age-groups	
<50 years (%)	14 (8.0%)
50-59 years (%)	36 (20.6%)
60-69 years (%)	74 (42.3%)
≥70 years (%)	51 (29.1%)
Aortic Primary Disease	
Stenosis (%)	143 (81.8%)
Regurgitation (%)	28 (15.9%)
Mixed	4 (2.3%)
NYHA	
NYHA II	68 (38.8%)
NYHA III	95 (54.3%)
NYHA IV	12 (6.9%)
Comorbities	
Arterial hypertension	133 (76.0%)
Type 2 diabetes	46 (26.3%)
Chronic kidney failure	8 (4.6%)
Carotid stenosis	8 (4.6%)
Chronic obstructive pulmonary disease	7 (4.0%)

**Table 2:** Baseline ultrasonography data BSA: Body surface area; ESC II: Euro score II; PsAP: Pulmonary artery systolic pressure

Baseline Ultrasonography Data	Mean ± Standard deviation	Minimal to maximal values
BSA (m^2^)	1.72 ± 0.15	1.30 – 2.10
Effective orifice area index (cm^2^/m^2^)	0.75 ± 0.15	0.49 – 1.11
Ejection Fraction (%)	60.9 ± 9.1	35.0 – 86.0
PsAP (mmHg)	42.7 ± 13.2	25 – 100
Annulus (mm)	19.9 ± 1.53	19 – 21
Posterior wall thickness (mm)	13.4 ± 2.15	10.0 – 22.0
Septum thickness (mm)	14.5 ± 2.37	8.0 – 23.0
Telediastolic diameter (mm)	49.3 ± 8.38	28.0 – 68.0
Telesystolic Diameter (mm)	33.1 ± 6.97	14.0 – 52.0
Maximal transvalvular aortic gradient (mmHg)	79.2 ± 25.46	24.0 – 160.0
Mean transvalvular aortic gradient (mmHg)	48.8 ± 15.70	14.0 – 103.0
ESC_II (%)	4.89 ± 2.98	0.83 – 18.93

A chi-square test was performed to compare low EOAi (≤ 0.85 cm^2^/m^2^) proportion between biological and mechanical aortic valve prosthesis (Table 3).

**Table 3:** Distribution based on the prosthesis type and presence of PPM EOAi: Indexed effective orifice area; p value<0.05 is significant/ Chi-square test

	EOAi ≤ 0.85 cm^2^/m^2^ (n = 133)	EOAi > 0.85 cm^2^/m^2^ (n = 42)	p - value
Biological valve prosthesis	64	3	<0.0001
Mechanical valve prosthesis	69	39	

**Figure 1:**
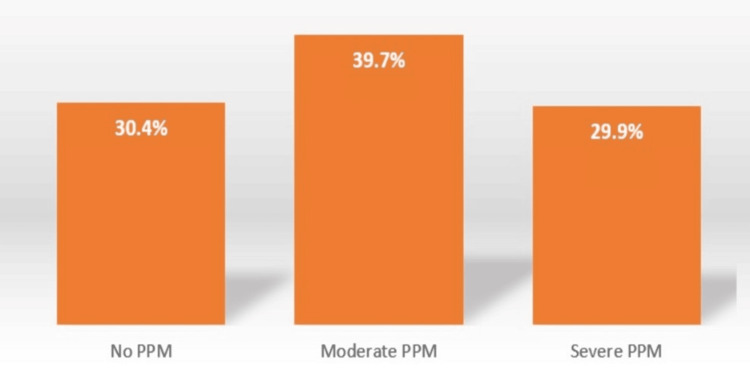
Mismatch severity across No- 19 valve replacement surgery PPM: Patient-prosthesis mismatch

**Figure 2:**
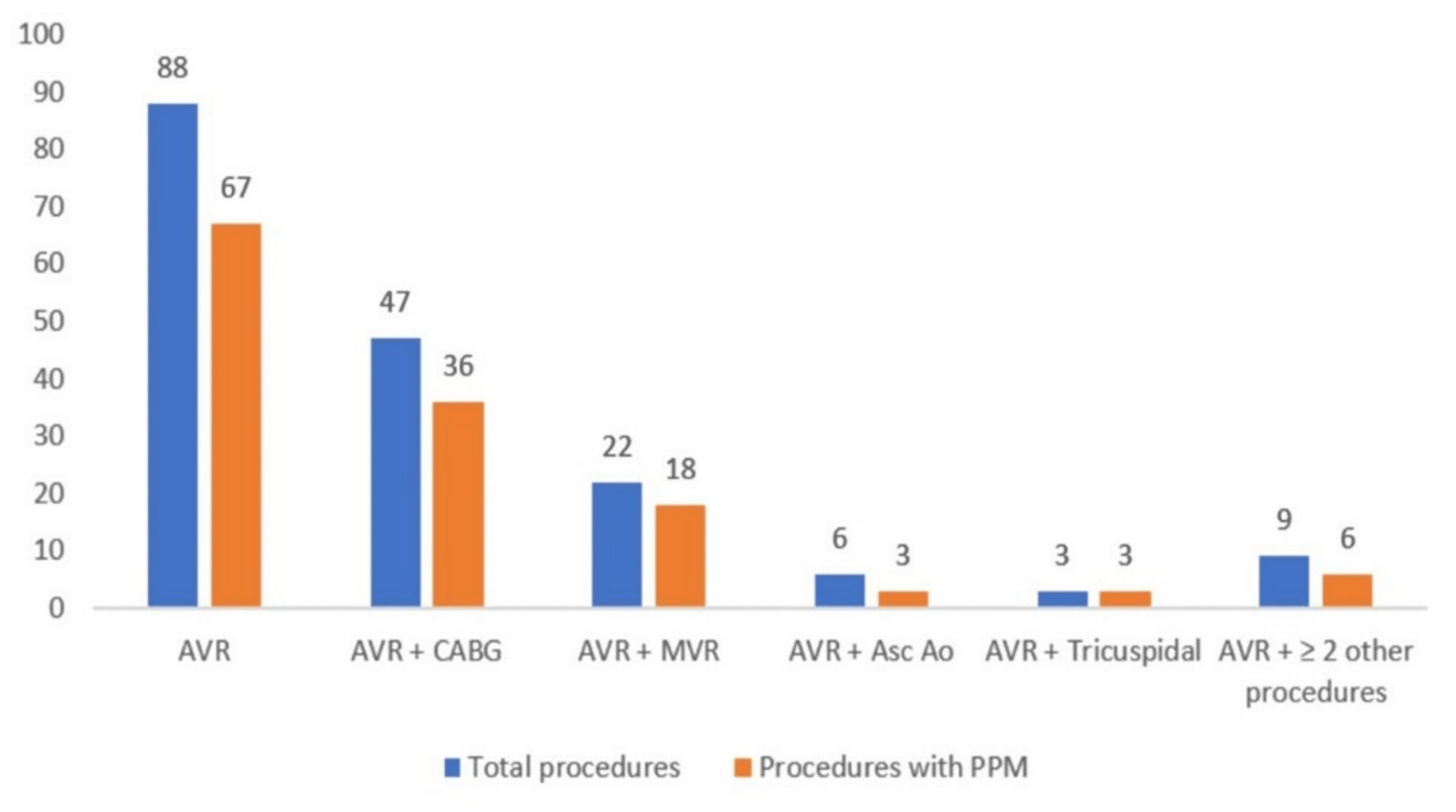
Distribution of mismatch across different surgical procedures AVR: Aortic valve Replacement; MVR: Mitral valve Replacement; Ao Asc: Ascendent aorta surgery; CABG: Coronary artery bypass grafting

The following graphs show the distribution of patient-prosthesis mismatch based on severity (Figure 1) and different surgical procedures (Figure 2).

Intraoperative and post operative times are as below: mean cardiopulmonary by-pass time 104.83±35.619 min, cross-clamp time 85.77±31.19 min, respiratory assistance time 15.35±30.37 hours intensive care unit stay time 60.09 ±34. Thirty two hours and total hospital stay of 9.7± 4.2 days (Table 4).

**Table 4:** Intraoperation and early operation period data CBP time: Cardio-pulmonary bypass time; ICU: Intensive Care Unit

Intraoperation and early operation period data	Mean ± Standard deviation	Minimal to maximal values
Clamp time (min)	85.8 ± 31.2	30.0 – 199.0
CBP time (min)	104.8 ± 35.6	41.0 – 240.0
ICU time (hours)	60.1 ± 34.3	20.0 – 217.0
Respiratory assistance (hours)	16.0 ± 30.4	2.0 – 308.0
Post-operative hospital stay (days)	9.8 ± 4.2	4.0 – 30.0

**Figure 3:**
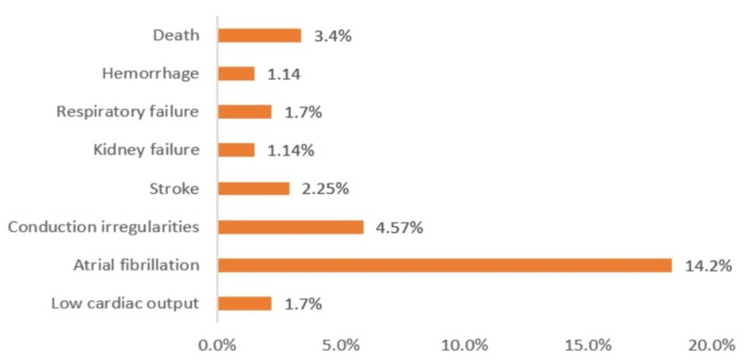
Incidence of early complications

Overall early mortality and major post-operative complications were as below: hospital mortality 3.4% (6/175), low cardiac output 1.7% (3/175); pulmonary complications 1.7% (3/175), hemorrhage 1.14% (2/175); atrial fibrillation 14.42% (25/175), conduction disturbances 4.57% (8/175), renal complications 1.14% (2/175) and stroke 2.25% (4/175) (Figure 3).

The following is a two-sample independent t-test that was performed to compare demographic, clinical, and surgical variables between the in-hospital deaths group and in-hospital survivors (Table 5).

**Table 5:** Association of demographic, clinical and surgical variables with short-term mortality BSA: Body surface area; CBP: Cardio pulmonary bypass time; EOAi: Indexed effective orifice area; PPM: Patient- prosthesis mismatch; CABG: Coronary artery bypass grafting; EF: Ejection Fraction (t-test. *Chi-square test; NS-not statistically significant)

	In-hospital deaths (n = 6)	In hospital survival (n = 169)	p-value
Age (years)	63.5	64.7	NS
Gender female (%)*	6 (100.0%)	121 (71.6%)	NS
BSA (m^2^)	1.712	1.715	NS
Clamp time (minutes)	144	83.9	0.049
CBP time (minutes)	158.2	103.1	0.038
EOAi (cm^2^/m^2^)	0.643	0.75	0.077
Severe PPM no(%)	4 (66.6%)	49 (29.0%)	0.048
CABG	2 (33.3%)	47 (27.8%)	NS
EF (%)	51.8	61.2	NS

**Table 6:** In-hospital mortality and EOAi association EOAi: Indexed effective orifice area; NS: Not statistically significant; logistic regression

Independent variable: EOAi	OR (Odds Ratio)	Confidence Interval 95%	p-value
EOAi – Basic Model	0.002	0.000 – 2.048	0.079
EOAi – Model I	0.000	0.000 – 26.114	NS
EOAi – Model II	0.000	0.000 – 73.309	NS

We created three models, as we have explained below, to detect the effect of PPM on early mortality. The statistical analysis results for the effect of EOAi as a continuous variable are shown in Table 6. The models showed the importance of PPM alone or between other negative predictors of early mortality (Table 7). The three models we created are: the basic model, model I, and model II. The basic model is adjusted for independent variables: age and gender. Model I is adjusted for independent variables: age, gender, clamp time, and extracorporeal circulation time. Model II is adjusted for independent variables: age, gender, clamp time, extracorporeal circulation time, CABG, and ejection fraction.

**Table 7:** In-hospital mortality and PPM association PPM: Patient-prosthesis mismatch; NS: not statistically significant; logistic regression

Independent variable: severe PPM	OR (Odds Ratio)	Confidence interval 95%	p-value
Severe PPM- Basic Model	5.6	0.95 – 33.6	0.057
Severe PPM – Model I	56.8	0.156 – 20677.8	NS
Severe PPM – Model II	1443.9	0.014 – 147281969.4	NS

The following tables present the long-term results of the statistical analyses (Table 8). During follow-up, 29 deaths occurred.

**Table 8:** Factors influencing long-term mortality BSA: Body surface area; CBP: Cardio pulmonary bypass time; EOAi: Indexed effective orifice area; PPM: Patient- prosthesis mismatch; CABG: Coronary artery bypass grafting; EF: Ejection Fraction (t-test ‘NS: not statistically significant; p value <0.05 is significant; (*)- borderline statistical significance.)

Variable	Mortality (29)	Survival (107)	p - value
Age (years)	68.0	62.5	0.001
Gender female (%)	9 (29.0%)	29 (26.9%)	NS
BSA (m^2^)	1.745	1.718	NS
Clamp time (minutes)	96.6	81.1	0.064
CBP (minutes)	114.4	100.6	NS
EOAi (cm^2^/m^2^)	0.695	0.765	0.019
Severe PPM (%)	13 (41.9%)	27 (25.0%)	0.066*
CABG	17 (54.8%)	19 (17.9%)	<0.001
EF (%)	62.5	60.8	NS

We followed the same method also for the long-term results with the main goal to detect the influence of EOAi (Table 9) as continuous variable and PPM as a categorical variable (Table 10) on the long-term mortality. 

**Table 9:** EOAi impact on long-term mortality EOAi: Indexed effective orifice area; NS: not statistically significant/ Logistic regression

Independent variable: EOAi	OR (Odds Ratio)	Confidence interval 95%	p - value
EOAi – Basic model	0.081	0.004 – 1.705	0.106
EOAi – Model I	0.111	0.001 – 9.024	NS
EOAi – Model II	0.064	0.000 – 12.222	NS

**Table 10:** PPM impact on long-term mortality PPM: Patient-prosthesis mismatch; NS: not statistically significant /Logistic regression

Independent variable: severe PPM	OR (Odds Ratio)	Confidence interval 95%	p - value
Severe PPM- Basic Model	1.71	0.72 – 4.05	NS
Severe PPM- Model I	1.76	0.53 – 1.12	NS
Severe PPM – Model II	3.48	0.72 – 16.49	NS

The analysis revealed that EOA-i and the PPM are not significant independent risk factors for late mortality in all analytic models we performed.

We analyzed the clinical status change, using NYHA class staging, before and after intervention as an important indicator for evaluating long-term results. A chi-square test was performed to compare NYHA class change groups with and without PPM (Table 11). A p-value of <0.05 was considered statistically significant. The presence of PPM negatively affects the improvement of clinical status. Three patient that were in PPM refer worsening of clinical situation after intervention. The group with PPM has a lower reduction of NYHA class (26.6%) in comparison with the group without PPM (64.7%).

**Table 11:** Patient's distribution based on NYHA class classification NYHA: New York Heart Association Classification (t-test; p value <0.05 is significant)

	EOAi ≤0.85 cm^2^/m^2^	EOAi > 0.85 cm^2^/m^2^	p - value
No. of patients	73 (56.4%)	34 (80.9%)	
NYHA class reduction	20 (26.6%)	22 (64.7%)	<0.0001
NYHA class advancement	3(4%)	0 (0%)	NS

We analyzed the NYHA change on three models, and the results show that severe PPM (Table 12) and EOAi (Table 13) are independent risk factors with high significance, respectively EOAi for Delta NYHA (HR=0.91; 95% CI: (-1.65)- (-0.18); p= 0.015) and severe PPM for Delta NYHA: (HR= -0.17; 95% CI: (-0.86)- (0.18) p= 0.186).

**Table 12:** Association of PPM with NYHA change during follow-up PPM: Patient-prosthesis mismatch; NS: Not statistically significant/ Linear regression; NYHA: New York Heart Association

Independent variable: Severe PPM	Beta	Confidence interval 95%	p-value
Severe PPM – Basic Model	-0.154	-0.050 – 0.359	0.37 (NS)
Severe PPM- Model I	-0.127	-0.083 – 0.336	0.232 (NS)
Severe PPM– Model II	-0.174	-0.086 – 0.434	0.186 (NS)

**Table 13:** Association of EOAi with NYHA change during follow-up PPM: Patient-prosthesis mismatch; p-value < 0.05 is significant/ Linear regression; EOAi: Indexed effective orifice valve area; NYHA: New York Heart Association

Independent variable: EOAi	Beta	Confidence interval (95%)	p-value
Severe PPM – Basic Model	1.000	-1.585 - -0.415	0.001
Severe PPM- Model I	0.865	-1.474 - -0.256	0.006
Severe PPM– Model II	0.919	-1.650 - -0.188	0.015

## Discussion

The prosthesis-patient mismatch can be described as a complication related to prosthesis selection. The small size of the prosthesis inherently limits the effective orifice area, leading to a higher likelihood of prosthesis-patient mismatch, particularly in patients with larger body surface areas. The influence on early and late outcomes of the aortic valve replacement remains an actual issue, even though this issue can be addressed at the moment of prosthesis implantation. Pibarot and Dumesnil state that the incidence of severe PPM varies from 2-11%, whereas that of moderate PPM is 20-70% [5]. The results from our study indicate a prevalence of 39.4% for moderate PPM and 29.7% for severe PPM. The presence of such a high frequency of severe PPM is explained by the fact that we have considered prosthesis no. 19. The impact of prosthesis-patient mismatch on both short-term and long-term outcomes remains a topic of debate among cardiothoracic surgeons due to conflicting findings from various international studies. The following is a discussion of international studies compared to the results of our study. When we discuss the results we obtained from our study, we should keep in mind that the main limitation of our study is the small study group and the patients who did not respond to the survey, as out of the initial 175 patients, only 136 of them responded to be includedin the long-term follow-up results.

Short-term mortality

The statistical analysis indicated that the factors influencing hospital mortality were clamping time, extracorporeal circulation time, EOAi, and severe PPM. Correlations on hospital mortality showed a weak association between mortality and EOAi (as a continuous variable) and severe PPM (as a categorical variable). This weak association is further reinforced by logistic regression for EOAi and severe PPM, where three models were considered. For the basic model, the association for both EOA-i and severe PPM is weak, while for the other two models (where the influence of more independent factors is added), the association is not significant.

Various international studies have concluded that early mortality is particularly increased in cases with severe PPM. The impact of severe PPM on early mortality is significant as the left ventricle is fragile, especially during the early post-operative period, and highly sensitive to even minor hemodynamic changes caused by the presence of severe PMM. Blais et al. (in a study that we also used and referenced for the tables of aortic prosthesis production companies) reported a hospital mortality rate of 4.6% during AVR. Severe PPM increases 11.4-fold the risk of mortality, while moderate PPM increases this risk to 2.1-fold. The group reported that the risk of mortality dramatically increased in patients with reduced left ventricle function, EF ≤ 40%, and severe PPM. The mortality rate in this group of patients was estimated at around 67%. This study also referred to lower mortality rates in the group of patients with preserved EF and mild to moderate PPM (2-5%) compared to 67% in the group of patients with reduced EF and severe PPM and 16% in patients with moderate PPM and reduced left ventricle function. Rao et al., in a study involving 2154 patients who underwent AVR, reported that the 30-day mortality rate was significantly higher in patients with evidence of PPM than in patients without PPM (7,9% versus 4,6%, p < 0.05). These results strengthen the idea that an insufficient ventricle is highly sensitive to increased afterload [6]. Dayan B and colleagues, in a very large meta-analytic study, yielded 382 articles for a total of 40,381 patients and found that perioperative and overall (i.e., perioperative and post-operative) mortality was increased in patients with PPM. Severe PPM was associated with an increased risk of both perioperative and overall mortality, whereas moderate PPM was associated with an increased risk of perioperative mortality but not of overall mortality [7]. These results suggest a connection between severe PPM and in-hospital mortality, but the limitations of our study, which are related to the small sample available, make it difficult to confirm this link. Larger studies are necessary. Nevertheless, there are reports that do not agree with the results mentioned above. Koene et al., from a study that included 2976 patients who underwent AVR and AVR+CABG, found that PPM was not an independent predictor for both early and late mortality [8].

Late/Long-term mortality

Regarding long-term mortality from statistical analysis, the chi-test resulted in a weak correlation between EOAi and severe mismatch. Logistic progression was done for each of these variables in three standardized models of independent factors; similar to short-term mortality, both variables (EOAi and severe PPM) lost significance. Therefore, as a conclusion of our statistical analysis, EOAi and severe PPM did not show significance in long-term mortality. Similar studies with small numbers of patients found no negative correlation between severe PPM and long-term survival of up to eight years [9,10]. Similar results were also found in a study by Milano et al. Freedom from sudden death (92%+/-5% vs 99%+/-1%, p = 0.01), valve-related death (84%+/-6% vs 90%+/-5%, p = 0.02), and cardiac events (56%+/-13% vs 86%+/-4%, p = 0.008) were importantly lower in a group with a higher incidence of PPM and also effective orifice area index was an independent predictor of late cardiac events. [11]. Tasca et al pointed out only severe PPM in patients with isolated aortic stenosis. It was revealed that 5-year survival and survival without cardiac events were 82% and 75% respectively in patients with severe PPM compared to 93% and 87% in those without severe PPM.Very similar results were reported for the eight-year survival study. Mechanical valves 19 and 21 were compared. An 8-year survival of around 41% was found in patients with severe PPM compared to 65% in those without severe PPM [12]. Another retrospective study by Mohty-Echahidi involved 388 patients who underwent AVR with No- 19 and 21 St. Jude prostheses. It also found that severe PPM is an independent predictor of higher long-term mortality and congestive heart failure in patients with small St Jude Medical aortic valve prostheses. [13] The Society of Thoracic Surgeons Adult Cardiac Surgery Database analyzed 59,779 patients over 65 years old who underwent isolated surgical aortic valve replacement between 2004 and 2014. Comparing patients with no PPM and patients with moderate or severe PPM had a significantly increased risk of rehospitalization for heart failure (hazard ratio [HR], 1.15; 95% confidence interval [CI], 1.09 to 1.21; HR, 1.37; 95% CI, 1.26 to 1.48) and redo AVR (HR, 1.41; 95% CI, 1.13 to 1.77; HR, 2.68; 95% CI, 2.01 to 3.56) for moderate or severe PPM, respectively. Survival was significantly lower for any degree of PPM (moderate to none: HR, 1.08; 95% CI, 1.05 to 1.12; severe to none: HR, 1.32; 95% CI, 1.25 to 1.39), with 10-year adjusted survival rates of 46%, 43%, and 35% for no PPM, moderate PPM, and severe PPM respectively (p < 0.001). Any degree of PPM affects importantly negatively long-term survival and increased rehospitalization rates for both heart failure and reoperation for AVR [14]. The reason for increased mortality and decreased longevity is mainly related to the fact that severe PPM negatively affects the regression rate of left ventricular mass, worsening post-operative hemodynamic performance over time and increasing the risk of heart failure.

Patients' clinical situation

A statistical analysis of data collected from a survey regarding patients' clinical outcomes revealed that 26.6% of patients with PPM had improvement in the NYHA class following the intervention, compared to 64.7% of those without PPM. Additionally, 4% of the PPM group showed a worsening of clinical status reflected by an increase in NYHA class compared to 0% of the non-PPM group.

Based on this finding, suspicion arose that EOAi and severe mismatch might influence patients' NYHA class change, thus affecting their clinical status and quality of life. 

In the correlation conducted for delta NYHA, all models showed that EOAi and severe PPM resulted in a strong correlation with NYHA class change. Therefore, small EOAi and severe PPM hinder NYHA class regression independently of other factors (age, gender, cross-clamping time, extracorporeal circulation time, CABG, ejection fraction, and pre-operative NYHA). (EOAi, p = 0.186; severe PPM, p = 0.015). The higher the mismatch, the smaller the change in NYHA class, and the larger the EOAi, the greater the clinical improvement. Analyses of delta NYHA show that factors become significant when compared to a larger sample of surviving patients than those who had passed away. Pibarot P et al. found that patients without PPM showed an improvement of +1.9 in the NYHA class compared to +1.5 in the PPM group. Non-regression of NYHA class reflects poor clinical progression of patients and may be due to the gradual development of heart failure over time, leading to the lack of regression in left ventricular mass or its slow regression and decrease in systolic function [15]. 

Sa et al. published a systematic review with meta-analysis that included 122989 patients with 592952 patient-years this year. At 25 years of follow-up, the survival rates were 11.8% and 20.6% in patients with and without any PPM (p<0.001). At 20 years of follow-up, the survival rates were 19.5%, 12.1%, and 8.8% in patients with no, moderate, and severe PPM, respectively (moderate versus no PPM: HR, p<0.001; severe versus no PPM (p<0.001). PPM was related significantly to a higher risk of cardiac death, heart failure-related hospitalizations, and aortic valve reinterventions over time (p<0.001). Significant associations between any degree of PPM and worse survival and heart failure as a cause of hospitalization were reported for both types of prosthesis, bioprosthetic, or mechanical valves [16]. Several studies do not support the idea that PPM negatively affects survival and clinical improvement. In 416 patients who received a mechanical 21 mm prosthesis, it was found that PPM or severe PPM does not impact long-term survival for up to 10 years in mechanical valve recipients when matched for pre-operative variables [17].

When we discuss biological prostheses, on the other hand, the majority of studies suggest that increasing grades of PPM are linked to poorer clinical outcomes in the long term (such as heart failure and mortality) in bio-prosthesis recipients [18,19].

Our study importantly found that PPM and smaller EOAi affect clinical improvement in the long term after aortic valve surgical replacement. This discussion makes sense because patient-prosthesis mismatch is a modifiable risk factor and is easily predictable. The operative team can predict and avoid PPM before implanting the prosthesis.

## Conclusions

The incidence and mismatch between prosthesis and patient in aortic valve replacement with a size 19 prosthesis are very high. The indexed effective orifice area and severe mismatch were found to be independent factors with low significance for early mortality but do not affect long-term survival. The indexed effective orifice area and severe mismatch significantly influence the clinical improvement in long-term follow-up. This emphasizes the need for optimal prosthesis selection and surgical technique to enhance patient-reported outcomes and overall quality of life.
